# High-flow nasal oxygen versus noninvasive ventilation in adult patients with cystic fibrosis: a randomized crossover physiological study

**DOI:** 10.1186/s13613-018-0432-4

**Published:** 2018-09-05

**Authors:** Michael C. Sklar, Martin Dres, Nuttapol Rittayamai, Brent West, Domenico Luca Grieco, Irene Telias, Detajin Junhasavasdikul, Michela Rauseo, Tai Pham, Fabiana Madotto, Carolyn Campbell, Elizabeth Tullis, Laurent Brochard

**Affiliations:** 1 Department of Anesthesia, University of Toronto, Toronto, Canada; 2 Interdepartmental Division of Critical Care Medicine, University of Toronto, Toronto, Canada; 3 Keenan Research Centre for Biomedical Science, Li Ka Shing Knowledge Institute, St. Michael’s Hopsital, 209 Victoria Street, 4th Floor, Room 411, Toronto, ON M5B 1T8 Canada; 4 Neurophysiologie Respiratoire Expérimentale et Clinique, Sorbonne Universités, Paris, France; 5 Division of Respiratory Diseases and Tuberculosis, Department of Medicine, Faculty of Medicine Siriraj Hospital, Bangkok, Thailand; 6 Division of Respirology, St. Michael’s Hospital, Toronto, Canada; 7 Department of Anesthesiology and Intensive Care Medicine, Catholic University of the Sacred Heart, Fondazione “Policlinico Universitario A. Gemelli”, Rome, Italy; 8 Department of Medicine, Faculty of Medicine Ramathibodi Hospital, Mahidol University, Bangkok, Thailand; 9 Department of Anaesthesia and Intensive Care, University of Foggia, Foggia, Italy; 10 Department of Medicine and Surgery, Research Center on Public Health, University of Milano-Bicocca, Monza, Italy

## Abstract

**Background:**

Noninvasive ventilation (NIV) is the first-line treatment of adult patients with exacerbations of cystic fibrosis (CF). High-flow nasal oxygen therapy (HFNT) might benefit patients with hypoxemia and can reduce physiological dead space. We hypothesized that HFNT and NIV would similarly reduce work of breathing and improving breathing pattern in CF patients. Our objective was to compare the effects of HFNT versus NIV in terms of work of breathing, assessed noninvasively by the thickening fraction of the diaphragm (TFdi, measured with ultrasound), breathing pattern, transcutaneous CO_2_ (PtcCO_2_), hemodynamics, dyspnea and comfort.

**Methods:**

Adult CF patients who had been stabilized after requiring ventilatory support for a few days were enrolled and ventilated with HFNT and NIV for 30 min in crossover random order.

**Results:**

Fifteen patients were enrolled. Compared to baseline, HFNT, but not NIV, reduced respiratory rate (by 3 breaths/min, *p* = 0.01) and minute ventilation (by 2 L/min, *p* = 0.01). Patients also took slightly larger tidal volumes with HFNT compared to NIV (*p* = 0.02). TFdi per breath was similar under the two techniques and did not change from baseline. MAP increased from baseline with NIV and compared to HFNT (*p* ≤ 0.01). Comfort was poorer with the application of both HFNT and NIV than baseline. No differences were found for heart rate, SpO_2_, PtcCO_2_ or dyspnea.

**Conclusions:**

In adult CF patients stabilized after indication for ventilatory support, HFNT and NIV have similar effects on diaphragmatic work per breath, but high-flow therapy confers additional physiological benefits by decreasing respiratory rate and minute ventilation.

**Clinical trial registration:**

Ethics Committee of St. Michael’s Hospital (REB #14-338) and clinicaltrial.gov (NCT02262871).

**Electronic supplementary material:**

The online version of this article (10.1186/s13613-018-0432-4) contains supplementary material, which is available to authorized users.

## Background

Cystic fibrosis (CF) is the most common autosomal recessive disorder in Caucasian populations [1]. Patients with CF experience exacerbations with hypercapnic respiratory failure associated with increased respiratory workload that may require intensive care unit (ICU) admission due to the inability of the respiratory muscles to compensate for an increased demand. Although no international recommendations currently exist [2], these exacerbations are frequently treated with noninvasive ventilation (NIV) [2, 3]. NIV has been shown to unload the respiratory muscles, increase alveolar ventilation and gas exchange [4] and reverse the rapid and shallow breathing pattern commonly adopted by CF patients with advanced lung disease [5].

NIV is a cornerstone therapy for hypercapnic acute respiratory failure [6, 7], but there is also an increasing interest in high-flow nasal therapy (HFNT) as a potential alternative treatment in this indication [8–10]. HFNT is a system delivering actively heated and fully humidified gas mixture with flow rates up to 60 L/min and adjustable FiO_2_ from 21 to 100%. The high flow rates generate small amounts of positive end-expiratory pressure (PEEP) that may help counterbalance the effects of intrinsic PEEP (PEEPi) on work of breathing and might act by washing out of the physiological dead space [11]. Furthermore, it could help to facilitate secretion clearance from the humidified gas [12].

Studies have demonstrated the benefits of HFNT in acute hypoxemic respiratory failure [13], after cardiothoracic surgery [14] and in the prevention of postextubation failure among unselected cohorts of critically ill patients during weaning from invasive mechanical ventilation [15, 16]. However, the current evidence of using HFNT in patients with hypercapnic acute respiratory failure is limited [9, 10, 17, 18]. CF is a unique respiratory disease with chronic, progressive, mixed obstructive–restrictive respiratory failure. From a physiological standpoint, this device could help CF patients with exacerbations in particular by improving gas exchange, reducing respiratory workload through reducing dead space, compensating for PEEPi and facilitating mucus clearance [19]. Additionally, a non-interrupted delivery of the technique with simple nasal prongs might improve comfort and tolerability compared to NIV via total face mask.

No study has compared the physiological effects of HFNT and NIV in adult CF patients requiring ventilator support. We hypothesized that HFNT and NIV would similarly reduce the work of breathing and improving breathing pattern in patients requiring ventilator support for CF exacerbations. Our objectives were to compare HFNT- and NIV-induced changes in inspiratory work of breathing assessed noninvasively by the thickening fraction of the diaphragm (TFdi) [20], breathing pattern, CO_2_ level, hemodynamics, dyspnea and comfort.

## Methods

Detailed methods are available in Additional file 1.

### Study design

**Fig. 1:**
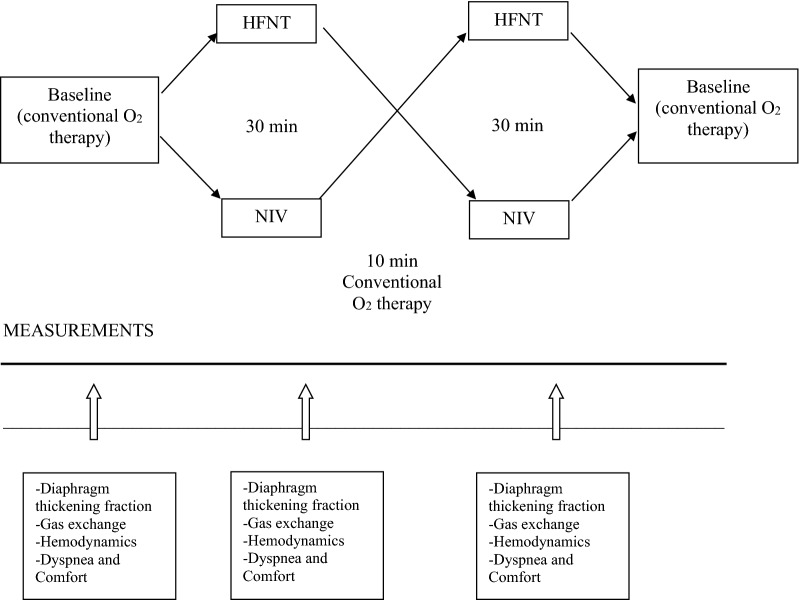
Study design. Fifteen cystic fibrosis patients were oxygenated with high-flow nasal cannula and noninvasive ventilation for 30 min each in random order with a 10-min washout period after each device use. All measurements were taken at baseline and after 25 min on each device. *HFNT* high-flow nasal therapy, *NIV* noninvasive ventilation

Physiological prospective randomized crossover study (Fig. 1), using noninvasive assessments, was approved by the Ethics Committee of St. Michael’s Hospital (REB #14-338) and registered on clinicaltrial.gov (NCT02262871). Patients provided their written informed consent. The study took place in a respiratory ward where NIV is usually delivered at St. Michael’s Hospital, Toronto, from January 2015 to February 2017.

### Patients

Hospitalized adult patients > 18 years of age with CF were included in the study if they had a clinical indication for NIV at the time of admission based on at least one of the following criteria [4, 21] and had been stabilized with NIV as set by respiratory therapists, medically optimized with antibiotics and bronchodilators as necessary according to their treating physician before entering the study (in order to minimize the risk of abrupt decompensation):

- Signs of clinical respiratory distress: respiratory rate > 24/min or accessory muscle use
- Partial pressure of arterial carbon dioxide (PaCO_2_) level > 45 mmHg from the time of hospital admission
- Nocturnal hypoventilation treated by NIV but requiring daytime NIV because of clinical worsening defined as either of the two conditions above
- Diurnal hypercapnia (PaCO_2_ > 45 mmHg) or PtcCO2 > 40 mmHg in patients who have persistent elevation of serum bicarbonate level (HCO_3_^−^ ≥ 32 mmol/L).

### Experimental procedure and study design

Enrolled patients received 30 min of HFNT or clinically set NIV in random order determined by a sealed opaque envelope. Physiological measurements were taken at baseline and at 25 min of each session of HFNT and NIV. Baseline oxygen settings were applied during a 10-minute washout period following the first device and at the end of the study.

HFNT (AIRVO-2^®^, Fisher & Paykel, Auckland, New Zealand) was set to a maximal inspiratory flow rate of 55 L/min as tolerated by the patient. FiO_2_ was adjusted to achieve a SpO_2_ of at least 92% and temperature at 37 °C or 34 °C according to patient’s individual preference.

Three NIV systems were used for the study depending on availability at enrollment (ResMed Stellar 150, ResMed VPAP III ST-A, ResMed Corp., California, USA or Respironics BiPAP Synchrony Respironics, Pennsylvania, USA). Two full face masks were available and were chosen based on patient preference: ResMed Hospital Full Face Mask (California, USA) and Fisher & Paykel Hospital Full Face Mask (Auckland, New Zealand). FiO_2_ was adjusted to achieve a SpO_2_ of at least 92%. NIV settings were those previously adjusted by the respiratory therapy team, based on oxygenation, arterial blood gases and patient tolerance; they were not modified for the purpose of the study and were kept unaltered during the study period.

### Data collection

Anthropometric data were obtained from the patient’s medical chart including age, gender, height and weight, and the most recent pulmonary function testing and arterial blood gasses. In addition, patient baseline characteristics including significant comorbidities, admission diagnosis and severity of illness (APACHE II) scores were collected.

### Physiological measurements

All patients were studied in the semi-recumbent position. Pulse oximetry and calibrated transcutaneous CO_2_ monitoring (SenTec Digital Monitoring System, Switzerland) were attached and monitored continuously during the study period [22]. A bio-impedance surface sensor (ExSpiron Monitor, Respiratory Motion, MA, USA) was placed and calibrated to measure noninvasively and continuously respiratory rate, tidal volume and minute ventilation [23].

A surrogate measure for work of breathing was estimated with diaphragm ultrasound as previously described [24, 25]. Diaphragm ultrasound examination was performed by a previously validated technique [26], using a SonoSite system (Fujifilm) equipped with a 13-MHz ultrasound linear probe. Diaphragm thickening fraction (TFdi) was calculated offline using the M mode [TFdi = (thickness at end inspiration − thickness at end expiration)/thickness at end expiration] with reviewers blinded to the mode of support.

Dyspnea and comfort scores were measured from 0 to 10 on a visual analog scale as described previously [27] and validated in patients with chronic obstructive pulmonary disease (COPD) [28]. (Dyspnea: 0 = no dyspnea, 10 = maximal dyspnea. Comfort: 0 = maximal discomfort, 10 = very comfortable.)

### Data management

Demographics, physiological data and recordings data were recorded and de-identified for confidentiality.

### Statistical considerations

At the time, this study was undertaken, there were no data on HFNT in hypercapnic patients, and nor was there robust physiological data on the use of NIV on adult patients with CF. In the absence of data allowing for an accurate estimation of sample size, we arbitrarily decided to enroll 15 patients in this exploratory study, with the hypothesis that this number would be sufficient to detect significant changes in respiratory effort.

Data are reported as median (interquartile range) for continuous variables and frequency (percentages) for categorical variables. The study is designed such that each participant is his/her own control. Differences between baseline conditions and each device as well as between devices differences were performed using the Wilcoxon signed-ranks paired test with a Bonferroni correction. Analyses were performed using Prism 4.01 software (GraphPad Software, San Diego, CA). For each comparison, a *p* value < 0.05 was considered significant.

## Results

### Patients

Fifteen adult patients with CF pulmonary exacerbations who had been stabilized over median 3 days under NIV and medical therapy were subsequently enrolled in the study (Additional file 1: Fig. 1). Patients were enrolled a median (interquartile range) of 5 (5–7) days after hospital admission. Characteristics of the subjects are detailed in Table 1. The NIV settings were those previously used by the clinicians and with which the patients had been stabilized. At baseline, all patients received oxygen via nasal prongs (range 0.5–10 L/min). All patients completed the study, and one patient did not have ultrasound measurements due to the unavailability of the machine at the time of data collection. In two patients, there were technical difficulties with the transcutaneous CO_2_ measurements. The ExSpiron monitor was not available in the early phase of the study and used for the latter 11 patients.

**Table 1:** Patient characteristics

Characteristics	*n* = 15
Female, *n* (%)	8 (53)
Age (years)	30 (23–34)
Body mass index (kg/m^2^)	19 (17–22)
APACHE II score at entry	8 (7–9.5)
*Baseline pulmonary function test*	
Forced expiratory volume in 1 s (L)	0.8 (0.6–1.0)
Forced expiratory volume in 1 s (% predicted)	24 (20–26)
Forced vital capacity (L)	1.49 (1.33–1.64)
FEV1/FVC ratio (%)	45 (42–52)
*Chronic comorbidities*	
Pancreatic insufficiency, *n* (%)	11 (73)
Diabetes mellitus, *n* (%)	5 (33)
Osteoporosis, *n* (%)	4 (27)
*Reason for admission*	
Cystic fibrosis exacerbation, *n* (%)	15 (100)
*Pulmonary therapy prior to admission*	
Supplemental oxygen as outpatient, *n* (%)	12 (80)
Noninvasive ventilation as outpatient, *n* (%)	9 (60)
Listed for lung transplantation, *n* (%)	10 (67)
*Arterial blood gases* ^a^	
pH	7.39 (7.38–7.41)
PaCO_2_ (mmHg)	53 (49–63)
HCO_3_^−^ (mmol/L)	34 (31–38)
PaO_2_ (mmHg)	64 (61–68)

Continuous variables are expressed as median (interquartile range), and categorical variables are expressed as absolute value (%)

^a^For arterial blood gas conditions, please see Additional file 1: Table 1

### Respiratory pattern at baseline

The median (interquartile range) baseline oxygen flow rate was 3 (2–5) L/min. Patients had a respiratory rate of 21 (17–26) breaths per minute and SpO_2_ of 93 (90–94) %. Dyspnea score was 1 (0–3) and comfort score 9 (8–10), confirming the clinical stabilization. TFdi was 30 (24–45) %.

### Effect of NIV and HFNT on respiratory pattern

HFNT flow rate was 45 (45–55) L/min with a FiO_2_ of 30 (25–35) %. NIV was set at an inspiratory positive airway pressure of 14 (12–18) cmH_2_O and a positive expiratory airway pressure of 6 (6–6) cmH_2_O. Flow rates, pressure settings and FiO_2_ were kept constant during the protocol. Individual device characteristics per patient are detailed in Additional file 1: Table 1.

**Fig. 2:**
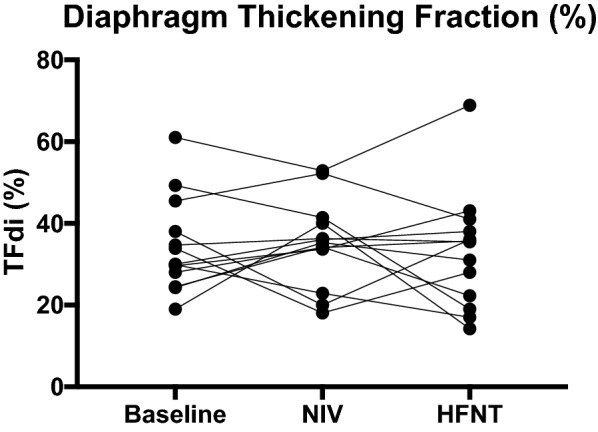
Individual patient changes in diaphragm thickening fraction. No significant change in diaphragm thickening fraction was observed between baseline conditions or after each device, *n* = 15

**Fig. 3:**
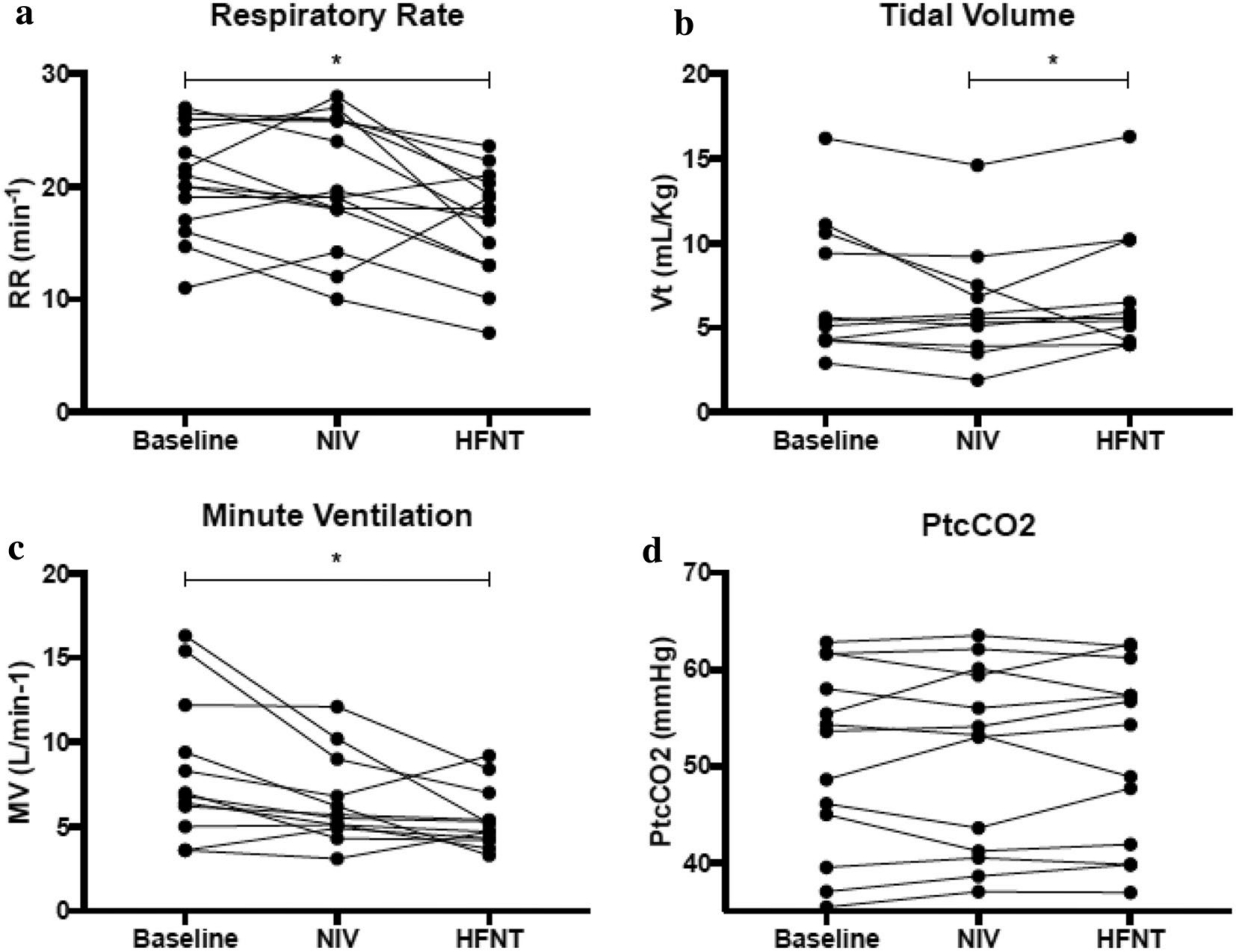
Individual patient changes in respiratory variables. **a** HFNT significantly reduced respiratory rate compared to baseline and **b** significantly increased tidal volume when compared to NIV, **c** HFNT significantly reduced minute ventilation compared to baseline conditions, **d** no differences were observed for PtcCO_2_, *p < 0.05 (versus baseline). *HFNT* high-flow nasal therapy, *NIV* noninvasive ventilation, *PtcCO*_*2*_ transcutaneous carbon dioxide, *n* = 15

No difference was observed in TFdi between baseline and either device (Table 2, Fig. 2). Compared to baseline, HFNT significantly reduced respiratory rate by a median (interquartile range) of 14% (8–37%), or 3 breaths/min, and minute ventilation by 27% (− 5 to 51%), or 2 L/min, (*p* = 0.01 for both) (Table 2, Fig. 3). Compared to NIV, HFNT slightly increased Vt by 10% (1.5–26%) (*p* = 0.02) (Table 2, Fig. 3). For pulse oximetry and PtcCO_2_, there was no change between baseline conditions and either device or between devices (Table 2 and Fig. 3).

**Table 2:** Changes in physiological variables induced by high-flow nasal cannula and noninvasive ventilation

Variables	Baseline	NIV	HFNT	*p* Baseline versus NIV	*p* Baseline versus HFNT	*p* NIV versus HFNT
*Respiratory pattern*						
Respiratory rate (min^−1^)	21 (17–26)	19 (18–26)	18 (13–20)	0.99	0.01	0.13
Tidal volume (mL/kg)	5.4 (4.3–10.6)	5.6 (3.9–10.6)	5.6 (4.2–10.2)	0.49	0.49	0.02
Minute ventilation (L/min)	6.9 (5.3–11.5)	5.6 (5.0–8.4)	5.0 (4.2–6.6)	0.46	0.01	0.46
SpO_2_ (%)	93 (90–94)	93 (92–94)	94 (93–95)	0.99	0.30	0.51
Transcutaneous CO_2_ (mmHg)	53 (42–60)	53 (41–60)	54 (41–60)	0.99	0.98	0.99
*Hemodynamic*						
Heart rate (min^−1^)	108 (89–114)	101 (92–115)	102 (91–114)	0.94	0.43	0.99
Mean arterial pressure (mmHg)	85 (78–91)	91 (81–101)	84 (77–94)	< 0.01	0.99	0.01
*Diaphragm activity*						
Thickening fraction (%)	30 (25–46)	35 (23–41)	36 (19–41)	0.99	0.99	0.99
*Symptoms*						
Dyspnea	1 (0–3)	1 (0–2)	1 (0–3)	0.82	0.99	0.99
Comfort	9 (8–10)	7 (6–9)	6 (5–8)	0.02	< 0.01	0.99

Continuous variables are expressed as median (interquartile range). Devices are compared head to head, and each device is compared to baseline conditions using the Wilcoxon signed-ranks test.

*HFNT* high-flow nasal therapy, *NIV* noninvasive ventilation, *n* = 15

In a sensitivity analysis, we removed one outlying patient (#14) with a large increase in TFdi with the application of NIV. This resulted in no changes to the statistical findings of our primary outcome of interest.

### Hemodynamics

NIV slightly but significantly increased MAP compared to baseline and HFNT conditions (*p* ≤ 0.01 for both) (Table 2). Heart rate remained unchanged across study conditions.

### Self-reported symptoms

**Fig. 4:**
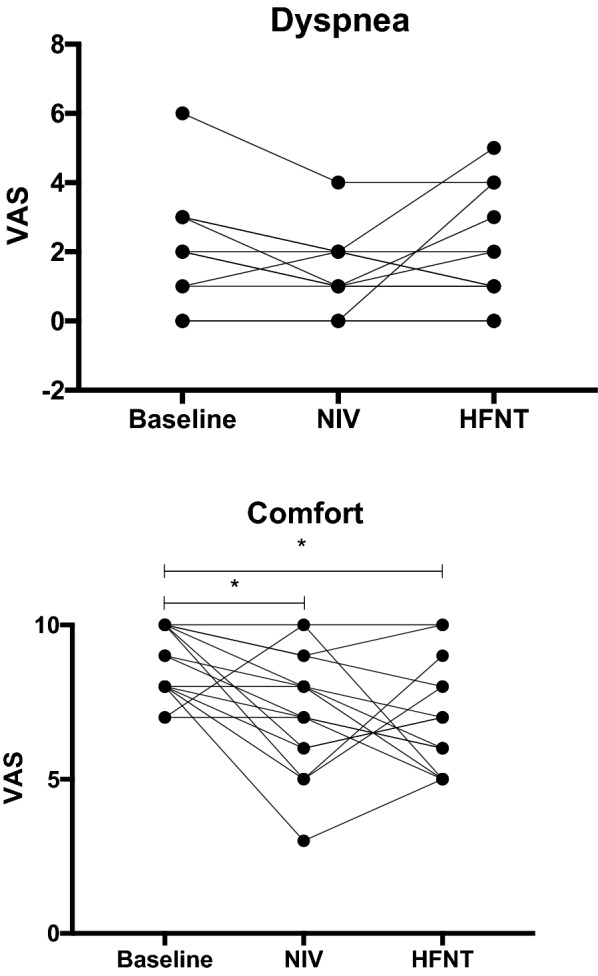
Individual patient changes in symptom scores, *n* = 15, with some symptom scores overlapping. **a** Dyspnea scores were unchanged between baseline conditions and the use of either HFNT or NIV. **b** Compared to baseline conditions, both NIV and HFNT reduced comfort scores, *p < 0.05 (versus baseline), *HFNT* high-flow nasal therapy, *NIV* noninvasive ventilation. Dyspnea and comfort scores: (dyspnea: 0 = no dyspnea, 10 = maximal dyspnea. Comfort: 0 = maximal discomfort, 10 = very comfortable), *n* = 15

Dyspnea scores were low, and there was no difference between baseline and any condition (median visual analog scale score of 1 for all three devices). Compared to baseline, however, comfort scores were significantly reduced with the application of both NIV and HFNT (9 (8–10) vs 7 (6–9) and 6 (5–8) (*p* = 0.02 and *p* < 0.01, respectively) (Table 2, Fig. 4). Thirteen out of the fifteen patients preferred one condition over the other, with 8 patients preferring NIV, while 5 had preference for HFNT.

## Discussion

To our knowledge, this is the first physiological study that specifically investigates the role of HFNT in adult CF patients who may have unique physiological responses compared to other forms of chronic respiratory failure secondary to inspissated secretions, mixed pulmonary mechanics and potentially altered structure and function of the diaphragm. The main result of this study is that diaphragmatic activity per breath, as assessed by TFdi, is similar between HFNT and clinically set NIV session, but that HFNT is associated with reduction in respiratory rate and minute ventilation implying a further reduction in total diaphragmatic work per minute with this technique.

No difference was observed in TFdi between HFNT and NIV, supporting our hypothesis, but we were surprised that there was no measurable significant reduction from either device compared to the baseline TFdi. This finding held true after removing one patient with outlying values, further strengthening our confidence in these results.

We thought it was essential to have all assessments and measurements taken with noninvasive techniques (e.g., external device for volume, ultrasonography for diaphragm activity, transcutaneous gas values) in order to minimize a direct effect of these techniques on the observed values (Heisenberg principle) [29, 30]. Importantly, however, these techniques are subject to measurement and performance error and we took great care to standardize the technical conditions for these measurements The lack of effect of diaphragmatic work might probably be explained by the relatively normalized (approximately 30%) TFdi in these patients at baseline [31, 32] had been stabilized before protocol implementation. Despite having other clinical and biochemical indicators for ventilatory support, our subjects had improved and been stabilized prior to protocol initiation (all subjects weaned to nasal prong oxygen), making it difficult to demonstrate a difference in diaphragmatic work of breathing. This is in contrast to a report of 14 stable chronically hypercapnic COPD patients in which both HFNT and NIV significantly reduced PTPdi (diaphragmatic work) [8]. Furthermore, in a more recent study including 5 hypercapnic patients recovering from acute respiratory failure, esophageal pressure swings, PTP and work of breathing (markers of respiratory effort) were significantly reduced with HFNT at 60 L/min [33]. This difference, however, may also stem from inherent differences in the structure and function of the diaphragm between COPD and CF patients [34].

It has been previously suggested that a concomitant metabolic alkalosis contributes to hypercapnic respiratory failure in exacerbations of adult CF and that these patients less frequently exhibit acidosis than COPD patients due to the abnormal electrolyte transport in CF patients compared to COPD, even during exacerbations [35, 36]. This may have been present in our subjects as well with a median pH in the normal range NIV which has been clinically used for hypercapnic respiratory failure in CF, but little is known about its physiological effects, particularly in the adult population. A Cochrane review on NIV in 191 mixed adult and pediatric CF patients for several indications was limited by small sample sizes of adult-only trials, sparse reporting of physiological variables and heterogeneous inclusion of varying degrees disease severity [21].

Consistent with previous work, HFNT demonstrated a significant reduction in respiratory rate [37]. Despite a statistically significant, but likely clinically inconsequential small increase in Vt, there was an overall substantial decrease in MV, which has been previously reported in COPD patients treated with HFNT [17]. This finding is partly explained by HFNT washing out of anatomical dead space, allowing a larger fraction of the MV to participate in gas exchange, increasing the efficiency of the respiratory system and decreasing the work of breathing [12]. It might also be related to decreases in nasal inspiratory resistance [38], which may lead to reduced PEEPi and inspiratory load [39]. Importantly, the reduction in respiratory rate might turn into a parallel decrease in the energy expenditure of the respiratory muscles, here by approximately 15%. Despite a significant reduction in minute ventilation, we found no concurrent reduction in PtcCO_2_, suggesting a reduced dead space and/or metabolic production. The significant reduction in respiratory rate and minute ventilation with HFNT suggests that the work of breathing per minute is substantially reduced, potentially between 15 and 25%, correlating with a reduction in CO_2_ production by the respiratory muscles [40].

We found no significant improvement in dyspnea scores. Comfort scores were significantly lower with both devices compared to baseline oxygen. This finding might reflect relatively stabilized patients with high baseline comfort scores. Moreover, this finding may represent the response to a short-term effect and could differ with a more prolonged use, as suggested in the literature [18, 41]. Oddly, 60% of patients were prescribed NIV at home prior to hospitalization, yet had lower comfort scores compared to baseline. Patients were accustomed to this treatment, even if they did not find it comfortable. We suspect this is another sign that patients were not anymore in distress.

This short-term physiological study has limitations. Firstly, epressure support levels during NIV were lower than compared to one NIV study performed in young patients with CF (11–18 cmH_2_O) [42]. These levels of pressure were not used clinically in our center, and this could have biased the results toward favoring a difference with HFNT. A recent Cochrane review, however, pointed out the paucity of studies in this situation to indicate optimal settings [21]. In addition, in a recent trial using NIV for physiotherapy, similar levels of pressure support (8 cmH_2_O) and PEEP (5 cmH_2_O) comparable to our study were used [43]. So, our data represent reasonable, although potentially not optimal, clinical settings. NIV was individually set according to clinical parameters and patient comfort by clinicians, allowing stabilization of the patients. This has probably resulted in normal levels of work of breathing. We are reassured that under NIV settings the TFdi was similar to values found in healthy volunteers [38]. Also, different NIV masks and NIV ventilators (based on resource availability) were used based on patient comfort. It is possible these different masks could perform differently with respect to the amount of leak, for example but the data obtained in other clinical settings do not suggest this has a great influence on work of breathing [44]. It is possible that, because 60% of our patients had used NIV at home, while none had used HFNT before, this could influence the physiological and subjective responses of our subjects.

Recent work suggests that physiological benefits of HFNT used in hypoxemic respiratory failure appear closely related to the applied flow rate [37]. Indeed, our median flow rate applied was 45 (45–55) L/min, and therefore, it is possible that further physiological improvement may have been seen if flow rates were maximized at 60 L/min. The level of flow rate, however, was dictated by patient’s preference. In addition, a recent study showed that most of the effect on inspiratory workload and CO_2_ clearance is already obtained at the lowest flow rate [45]. In addition, it remains unclear whether the relationship between flow rate and physiological response holds true in chronically hypercapnic patients.

In contrast to previous physiological studies on HFNT [8, 33, 37] that used esophageal balloon catheters, we chose to utilize diaphragm ultrasonography as our primary means of estimating patient work of breathing as previously demonstrated [24]. Although our approach makes direct comparison with these previous studies more difficult, we feel that diaphragm ultrasound has become a well-studied modality, with described reproducibility, applied noninvasively and relatively easily learned [43]. Previous work has characterized the technical performance of diaphragm ultrasonography with thickness measurements being highly reproducible (mean ± SD 2.4 ± 0.8 mm, repeatability coefficient 0.2 mm, reproducibility coefficient 0.4 mm), while thickening fraction was only moderately reproducible (median 11%, IQR 3–17%, repeatability coefficient 17%, reproducibility coefficient 16%) [43].

Sputum production and tenacity is a hallmark feature of CF; however, we did not assess changes in secretion characteristics during this study. Although the added humidity of HFNT could reduce secretion thickness, this should be an area of future investigation.

An important limitation was that patients were stabilized prior to study inclusion, as already mentioned. Importantly, however, we demonstrated that HFNT significantly improves breathing pattern in patients recovering from CF exacerbations and therefore holds promise that HFNT may confer benefit even in more acute and decompensated presentations.

## Conclusion

No difference is observed in HFNT compared to NIV with respect to the diaphragmatic work per breath in CF patients stabilized after a clinical indication for ventilatory support but significantly reduces the respiratory rate and the work per minute. These preliminary data suggest that high-flow therapy may confer physiological benefits by decreasing ventilation needs and may constitute an interesting alternative or supplement to NIV.

## Additional file

**Additional file 1.** Electronic supplementary material.

## Abbreviations

COPD: chronic obstructive pulmonary disease
CF: cystic fibrosis
HFNT: high-flow nasal therapy,
ICU: intensive care unit
NIV: noninvasive ventilation
PEEP: positive end-expiratory pressure
PEEPi: intrinsic positive end-expiratory pressure
PaCO_2_: partial pressure of arterial carbon dioxide,
PtcCO_2_: transcutaneous carbon dioxide
PTPdi: pressure time product of the diaphragm
TFdi: thickening fraction of the diaphragm
