# Supplementary material for: High-flow nasal oxygen versus noninvasive ventilation in adult patients with cystic fibrosis: a randomized crossover physiological study
Source: Ann Intensive Care. 2018 Sep 5;8:85. doi: 10.1186/s13613-018-0432-4 (PMC6125258; doi:10.1186/s13613-018-0432-4)
Supplement: Supplementary file 1 — Additional file 1. Electronic supplementary material. [file 13613_2018_432_MOESM1_ESM.docx]

**High Flow Nasal Oxygen vs. Non-Invasive Ventilation in Adult Cystic Fibrosis Patients: A Randomized Cross-Over Physiological Study**

**Supplementary Appendix**

Michael C Sklar^1,2^ MD, Martin Dres^3,4^ MD, PhD, Nuttapol Rittayamai^3,5^MD, Brent West^6^ RRT, Domenico Luca Grieco^3,7^ MD, Irene Telias^2,3^ MD, Detajin Junhasavasdikul^3,8^ MD, Michela Rauseo^2,9^ MD, Tai Pham^2^ MD, PhD, Fabiana Madotto^10^, Carolyn Campbell^3^, Elizabeth Tullis^6^ MD, Laurent Brochard^2,3^ MD, PhD

**INSTITUTIONS.**

*^1^Department of Anesthesia, University of Toronto, Toronto, Canada*

*^2^ Interdepartmental Division of Critical Care Medicine, University of Toronto, Toronto, Canada*

*^3^ Keenan Research Centre, Li Ka Shing Knowledge Institute, St. Michael's Hospital, Toronto, Canada
^4^Neurophysiologie Respiratoire Expérimentale et Clinique, Sorbonne Universités, Paris, France*

*^5^ Division of Respiratory Diseases and Tuberculosis, Department of Medicine, Faculty of Medicine Siriraj Hospital, Bangkok, Thailand*

*^6^ Division of Respirology, St. Michael's Hospital, Toronto, Canada*

*^7^ Department of Anesthesiology and Intensive Care Medicine, Catholic University of the Sacred Heart, Fondazione “Policlinico Universitario A. Gemelli”, Rome, Italy*

*^8^ Department of Medicine, Faculty of Medicine Ramathibodi Hospital, Mahidol University, Bangkok, Thailand*

*^9^ Department of Anaesthesia and Intensive Care, University of Foggia, Foggia, Italy*

*^10^ Research Center on Public Health, Department of Medicine and Surgery, University of Milano-Bicocca, Monza, Italy*

**Materials and Methods**

*Non-Inclusion Criteria*

Patients were not eligible for enrollment if they had any of the following: active massive hemoptysis, pneumothorax with pleural drainage and persistent air leak, hemodynamic instability requiring vasopressors, uncooperative behavior, recent upper airway or esophageal surgery, skin or chest wall or abdominal trauma (that prevents placement of the transducer band), declared pregnancy. All patients received standard therapy (antibiotics, nebulizer treatments) at the discretion of the attending physician.

*Physiological Measurements*

*The ExSpiron Monitor*

The ExSpiron monitor is applied and calibrated as follows: 1 electrode pad comprising 3 electrodes was placed along the sternum and the other electrode pad comprising 3 electrodes was placed across the right midaxillary line at the level of the xiphoid process [1]. To calibrate the system, patients performed one minute of breathing into a Wright spirometer (nSpire Health, Inc., Longmont, CO) collected through a single-use mouthpiece and filter while wearing a disposable nose clip. The generated minute volume was then input into the ExSpiron system along with patient height and weight for calibration.

*Diaphragmatic Ultrasonography*

Ultrasound measurements were performed by MCS, MD, DLG, DJ and NR. All sonographers were extensively trained in this technique by an internal validation process (>15 independent and validated evaluations). To improve reproducibility in measurements, a skin marker was used at the beginning of the protocol to identify the area to re-measure during subsequent examinations during the protocol as recommended [2]. Diaphragm thickness was measured both at end-inspiration and end-expiration at tidal breathing, by placing the transducer in the right eighth or ninth intercostal space between the anterior and mid-axillary line and directing the ultrasound beam perpendicular to the diaphragm. The measurements were performed using the caliper function on the ultrasound machine and thickening fraction was averaged over at least four breaths in M-mode.

1. Williams GW, George CA, Harvey BC, Freeman JE. A Comparison of Measurements of Change in Respiratory Status in Spontaneously Breathing Volunteers by the ExSpiron Noninvasive Respiratory Volume Monitor Versus the Capnostream Capnometer. Anesth Analg. 2017;124:120–6.

2. Goligher EC, Laghi F, Detsky ME, Farias P, Murray A, Brace D, et al. Measuring diaphragm thickness with ultrasound in mechanically ventilated patients: feasibility, reproducibility and validity. Intensive Care Med. 2015;41:642–9.

**Figure Legends**

**Figure 1:** Study flow. HFNT – high flow nasal therapy, NIV – non-invasive ventilation.

**Table 1. Device Characteristics**

| **Patient** | **Baseline Oxygen (L/min)** | **NIV IPAP (cmH_2_O)** | **NIV EPAP (cmH_2_O)** | **HFNT Flow (L/min)** | **HFNC FiO_2_ (%)** |
| --- | --- | --- | --- | --- | --- |
| 1 | 2 | 11 | 6 | 55 | 25 |
| 2 | 2 | 14 | 6 | 55 | 21 |
| 3 | 2 | 18 | 6 | 45 | 28 |
| 4 | 2 | 12 | 6 | 45 | 28 |
| 5 | 3 | 15 | 5 | 45 | 32 |
| 6 | 1 | 14 | 6 | 55 | 28 |
| 7 | 10 | 12 | 6 | 45 | 40 |
| 8 | 8 | 19 | 7 | 50 | 50 |
| 9 | 4 | 22 | 6 | 55 | 32 |
| 10 | 3 | 18 | 6 | 45 | 30 |
| 11 | 5 | 16 | 6 | 50 | 35 |
| 12 | 4 | 14 | 8 | 45 | 32 |
| 13 | 5 | 14 | 6 | 45 | 40 |
| 14 | 1.5 | 20 | 6 | 50 | 25 |
| 15 | 0.5 | 12 | 6 | 45 | 21 |
| Median (IQR) | 3 (2-5) | 14 (12-18) | 6 (6-6) | 45 (45-55) | 30 (25-35) |

Baseline oxygen was delivered via nasal prongs.

EPAP – expiratory positive airway pressure, FiO_2_ – fraction of inspired oxygen, IPAP – inspiratory positive airway pressure, HFNT – high flow nasal therapy

**Figure 1: Study Flow**
